# Self-Reported DHA Supplementation during Pregnancy and Its Association with Obesity or Gestational Diabetes in Relation to DHA Concentration in Cord and Maternal Plasma: Results from NELA, a Prospective Mother-Offspring Cohort

**DOI:** 10.3390/nu13030843

**Published:** 2021-03-04

**Authors:** Antonio Gázquez, María J. Giménez-Bañón, María T. Prieto-Sánchez, Carmen Martínez-Graciá, Clara Suárez, Marina Santaella-Pascual, Lina Galdo-Castiñeira, Carmen Ballesteros-Meseguer, Jesús Vioque, Miriam Martínez-Villanueva, Francisco Avilés-Plaza, José A. Noguera-Velasco, Eva Morales, Luís García-Marcos, Elvira Larqué, on behalf of the Nela Study Group

**Affiliations:** 1 Biomedical Research Institute of Murcia (IMIB-Arrixaca), 30120 Murcia, Spain; antonio.gazquez@um.es (A.G.); gimenezba@hotmail.com (M.J.G.-B.); mt.prieto@um.es (M.T.P.-S.); mamen@um.es (C.M.-G.); clara.suarez@um.es (C.S.); marinasp@um.es (M.S.-P.); miriam.martinez3@um.es (M.M.-V.); franciscovaleriano.aviles@um.es (F.A.-P.); joseantonio.noguera@um.es (J.A.N.-V.); evamorales@um.es (E.M.); lgmarcos@um.es (L.G.-M.); 2 Department of Physiology, University of Murcia, 30100 Murcia, Spain; 3 Obstetrics & Gynaecology Service, “Virgen de la Arrixaca” University Clinical Hospital, University of Murcia, 30120 Murcia, Spain; linagaldo@hotmail.com (L.G.-C.); carmenbm@um.es (C.B.-M.); 4 Food Science and Technology Department, Faculty of Veterinary, University of Murcia, 30100 Murcia, Spain; 5 Health and Biomedical Research Institute of Alicante (ISABIAL-UMH), 46020 Alicante, Spain; vioque@umh.es; 6 CIBER Epidemiology and Public Health (CIBERESP), 28029 Madrid, Spain; 7 Molecular Therapy and Biomarkers Research Group, Clinical Analysis Service, University Clinical Hospital “Virgen de la Arrixaca”, University of Murcia, 30120 Murcia, Spain; 8 Department of Public Health Sciences, University of Murcia, 30100 Murcia, Spain; 9 Network of Asthma and Adverse and Allergic Reactions (ARADyAL), 28029 Madrid, Spain

**Keywords:** obesity, docosahexaenoic acid, diabetes, pregnancy, supplementation, fatty acids

## Abstract

Maternal supplementation of docosahexaenoic acid (DHA) during pregnancy has been recommended due to its role in infant development, but its effect on materno-fetal DHA status is not well established. We evaluated the associations between DHA supplementation in pregnant women with obesity or gestational diabetes mellitus (GDM) and maternal and neonatal DHA status. Serum fatty acids (FA) were analyzed in 641 pregnant women (24 weeks of gestation) and in 345 venous and 166 arterial cord blood samples of participants of the NELA cohort. Obese women (*n* = 47) presented lower DHA in serum than those lean (*n* = 397) or overweight (*n* = 116) before pregnancy. Linoleic acid in arterial cord was elevated in obese women, which indicates lower fetal retention. Maternal DHA supplementation (200 mg/d) during pregnancy was associated with enhanced maternal and fetal DHA levels regardless of pre-pregnancy body mass index (BMI), although higher arterial DHA in overweight women indicated an attenuated response. Maternal DHA supplementation was not associated with cord venous DHA in neonates of mothers with GDM. The cord arteriovenous difference was similar for DHA between GDM and controls. In conclusion, maternal DHA supplementation during pregnancy enhanced fetal DHA status regardless of the pre-pregnancy BMI while GDM may reduce the effect of DHA supplementation in newborns.

## 1. Introduction

Docosahexaenoic acid (DHA) is an omega-3 long-chain polyunsaturated fatty acid (LC-PUFA) that rapidly accumulates on human brain during the last trimester of pregnancy and the first months of life [1,2]. An appropriate supply of DHA before delivery is of special relevance for visual and cognitive development of the newborn and child [3,4]. The main source of DHA for the fetus is the transfer of the DHA from maternal circulation across the placenta [5]. However, dietary DHA intake is inadequate in many pregnant women (less than 1–2 portions of oily fish per week) and therefore, maternal dietary supplementation of at least 200 mg DHA/d during pregnancy and lactation is recommended [6,7].

Some recent studies reported that pre-pregnancy body mass index (BMI) was inversely associated with polyunsaturated fatty acids (PUFA), DHA, and omega-6 fatty acids (FA) in venous cord blood while the results were contradictory in the mothers [8,9]. Similarly, gestational diabetes mellitus (GDM) has been associated with lower proportion of AA (arachidonic acid, 20:4 omega-6) and DHA in umbilical vein plasma but not in maternal plasma [10,11]. Disturbances on placental FA transport using labeled FA with stable isotopes [12,13] and altered FA transporters protein expression in placental tissue of obese and GDM women have been reported [14,15,16]. These alterations may affect the efficacy of maternal DHA supplementation during pregnancy for improving neonatal DHA status at birth.

In addition, an altered handling/metabolism of LC-PUFA in fetus from GDM mothers have been proposed by some authors since they found lower DHA in umbilical artery plasma in GDM than in controls but not in umbilical vein plasma [17,18]. However, there are no large studies that have evaluated both arterial and venous FA profile to confirm this hypothesis and not one among obese pregnant women. Both lower PUFA placental transport and altered fetal handling of these essential micronutrients could affect the efficiency of DHA supplementation in these pregnancies and should be investigated in healthy and pathological pregnancies.

Supplementation of GDM women during pregnancy with 600 mg/d DHA enhanced maternal but not fetal DHA status in a randomized double-blinded placebo-controlled trial, suggesting that placental tissue in GDM could reduce the effect of DHA supplementation in the fetus [19]. Monthe-Dreze et al. investigated the effect of DHA supplementation in obese women; in all BMI groups, pregnant women had higher omega-3 concentrations following supplementation, although obese women had attenuated changes (β = −2.04, CI: −3.19–−0.90, interaction *p* = 0.000) compared to lean women, resulting in a 50% difference in the effect size [20]. To our knowledge the effect of maternal DHA supplementation on women with obesity on their offspring DHA status has not been evaluated yet. Better knowledge of PUFA metabolism may help to improve supplementation guidelines and hence both mother and offspring health outcomes.

The aim of the present study is to evaluate the associations between DHA supplementation in obese pregnant women with obesity or GDM and fetal DHA status in a prospective mother–child cohort study in Spain. As secondary objective, we want to explore whether the differences in PUFA fetal levels are due to an altered placental transport and/or to an impairment of fetal metabolism through the study of the FA arteriovenous cord concentrations.

## 2. Materials and Methods

### 2.1. Study Participants

Participants from the Nutrition in Early Life and Asthma (NELA) study (www.nela.imib.es, accessed on 15 December 2020), a prospective population-based birth cohort set up in Murcia, a south-eastern Mediterranean region of Spain, were included [21]. The main objective of NELA cohort is to unravel the developmental origins and mechanisms of asthma and allergy.

Pregnant women who fulfill the inclusion criteria were invited to participate in the study at 20 weeks of gestation in the Maternal-Fetal Unit of the Virgen de la Arrixaca University Hospital, from March 2015 to April 2018. The inclusion criteria were as follows: Women from Health Area I and certain districts of Health Areas VI and VII of the Region of Murcia, planning to live in the area of study during at least 2 years and intention to give birth at the reference hospital; Caucasian origin; 18–45 years of age; singleton pregnancy; spontaneous conception; and normal echography at 20 weeks of gestation (no major malformations). The exclusion criteria were: An existing chronic disease and pregnancy complications, except GDM and hypertensive disorders.

**Figure 1:**
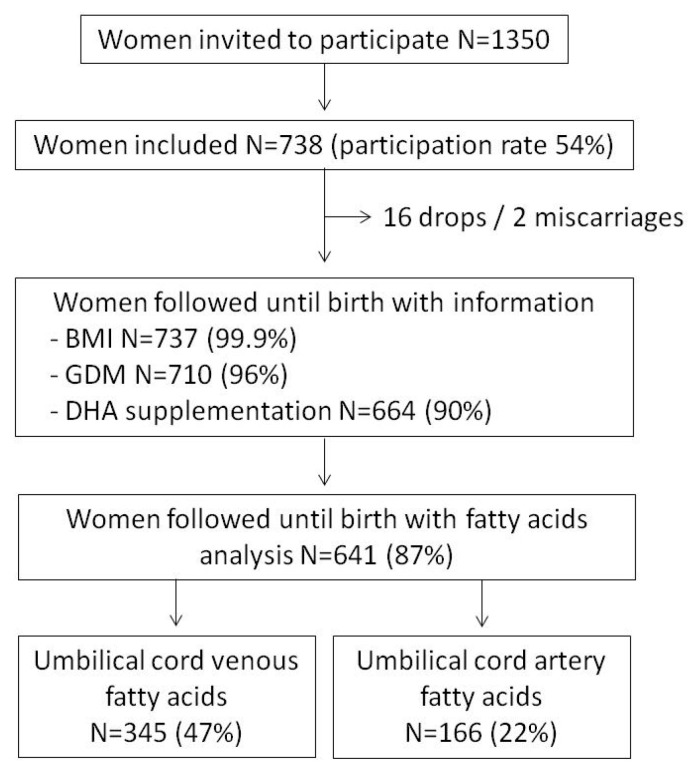
Flow diagram of the study.

Among the 1350 women invited to participate, 738 (54%) were finally enrolled in the study and 664 (90%) had data on DHA supplementation during pregnancy and 641 (87%) FA analysis at mid-pregnancy (Figure 1). Umbilical cord venous FA were analyzed in 345 (47%) subjects and only 166 (22%) samples of cord artery were quantified. According to the self-reported pre-pregnancy BMI, 456 women were classified as lean (BMI 18.5–24.9 kg/m^2^, 62%), 145 overweight (BMI 25–29.9 kg/m^2^, 20%) and 62 obese (BMI > 30 kg/m^2^, 8%).

GDM was diagnosed with O’Sullivan test between 24–28 weeks of gestation by screening with 50 g oral glucose. A positive result (1 h plasma glucose > 140 mg/dL) was followed by a 3 h oral glucose test with 100 g glucose. The test was considered positive with two or more glucose values were above the cut off according to the National Diabetes Data Group Criteria [22].

The study protocol was reviewed and approved by the Ethics Committee of the Virgen de la Arrixaca University Clinical Hospital in accordance with the guidelines of The Declaration of Helsinki. Written informed consents were obtained from parents at recruitment.

### 2.2. Maternal and Neonatal Anthropometrical Measurements

Maternal pre-pregnancy BMI was calculated based on height measured by trained personnel of the cohort and pre-pregnancy self-reported weight (kg/m^2^) and categorized as normal (18.5–24.9 kg/m^2^), overweight (25–29.9 kg/m^2^), and obese (≥30 kg/m^2^). Maternal weight during pregnancy was measured by trained personnel at 20 and 32 weeks of gestation. Gestational weight gain during pregnancy was self-reported by the participants at delivery.

Anthropometrical variables of the neonate including birthweight (kg), length (cm), and head circumference (cm) were obtained from clinical records. Newborn BMI and the z-score were calculated using Spanish reference data [23]. Nutritional status of the newborn was calculated from z-score according to the World Health Organization: Low birth weight (<−2SD), normal weight (−2SD–+1SD), overweight risk (>+1SD), overweight (>+2SD), and obese (>+3SD) [24].

### 2.3. Assessment of Maternal DHA Intake from Diet and Use of Supplements during Pregnancy

Maternal diet during gestation was assessed at 20 weeks of gestation using a validated food frequency questionnaire [25] administrated by trained interviewers. Nutrient values, including DHA intake, were primarily obtained from food composition tables from the US Department of Agriculture [26], and other published sources reporting information on FA content in Spanish foods [26,27,28,29,30]. We calculated the usual daily nutrient intakes for each woman by multiplying the frequency of the use of each food item by the nutrient content of the portion size specified in the food frequency questionnaire. Then we added all foods to obtain the total nutrient intake for each participant. The usual daily intake of DHA was expressed in mg/day. Furthermore, we estimated the energy intake in kcals/day for each participant. We used the residual method to estimate calorie-adjusted values for the nutrient intakes [31]. Information on the use of DHA supplements was collected by questionnaires at 20 and 32 weeks of gestation. Daily dose of DHA intake from supplements was estimated based on supplement brand name and composition.

### 2.4. Sampling

Ten milliliters of maternal blood at 24 weeks of gestation and 1 mL each of both venous and arterial cord blood were collected at delivery. Serum was separated by centrifugation at 1400 g for 5 min within 1 h from delivery. Glucose was measured using an automatic analyzer (Roche-Hitachi Modular PyD Autoanalyzer, Mannheim, Germany). Serum was frozen at −80 °C until FA analysis.

### 2.5. Fatty Acid Quantification in Maternal and Umbilical Cord Serum

Total lipids were extracted from 100 μL serum into chloroform:methanol (2:1 *v/v*) according to Folch et al. method [32]. Previous to the extraction, 0.05 mg pentadecanoic acid was added to the samples as internal standard. FA methyl esters were produced according to Stoffel et al. [33] by adding 1 mL of 3 *n* methanolic HCl (Supelco, Sigma-Aldrich, St. Louis, MO, USA) and heating at 90 °C for 1 h. The derivatives were extracted into hexane and stored at −20 °C until gas chromatographic analysis.

FA methyl esters were analyzed by gas chromatography using a SP-2560 capillary column (100 m × 0.25 mm i.d. × 20 µm) (Supelco, Sigma-Aldrich, St. Louis, MO, USA) in a Hewlett-Packard 6890 gas chromatograph (Agilent Technologies, Santa Clara, CA, USA) equipped with a flame ionization detector. The temperature of the detector and the injector was 240 °C. The oven temperature was programmed at 175 °C 30 min and increased at 2 °C/min to 230 °C and held at this temperature for 17 min. Helium was used as the carrier gas at a pressure of 45 psi. ChemStation software (Agilent Technologies, Santa Clara, CA, USA) was used to analyze FA data. Peaks were identified by comparison of their retention times with appropriate FA methyl esters standards (Sigma-Aldrich, St. Louis, MO, USA) and FA concentrations determined in relation to peak area of internal standard.

### 2.6. Covariates

We obtained information through questionnaires administered in person during pregnancy about maternal age, parity (0, 1 and >1), time from last pregnancy (<12 months vs. ≥12 months), maternal educational level (incomplete secondary or less, complete secondary and university), maternal social class (defined as maternal occupation during pregnancy by using a widely used Spanish adaptation of the international ISCO88 coding system: I–II, managers/technicians; III, skilled; IV–V, semiskilled/unskilled; and unemployed) [34], maternal smoking during pregnancy (yes/no), and maternal alcohol use (yes/no).

Information related to newborn’s sex, gestational age at delivery (weeks), pre-eclampsia (yes/no), and mode of delivery (eutocic vaginal delivery, instrumented vaginal delivery, and caesarean section) was collected form clinical records.

### 2.7. Statistical Analysis

Results were expressed as mean ± standard deviation for normal variables and absolute frequencies or percentages for those categorical. Sociodemographic characteristics between the three pre-pregnancy BMI groups were analyzed using one-way ANOVA followed by a post hoc Bonferroni test and for non-parametric variables Kruskal–Wallis test. For non-quantitative variables Chi-Square test was used. Univariate general linear models ANCOVA were used to analyze the results on FA by maternal BMI or GDM adjusted by potential confounders. Confounders were evaluated by linear regression analyses (Figure S1).

The associations between DHA levels and maternal/neonatal variables were analyzed using multiple linear regression analyses and corrected by potential confounders. Logistic regression models were applied to calculate the odds ratios (OR) and 95% confidence intervals (CI) of having different response to dietary DHA supplements depending on maternal BMI or GDM condition, also adjusted for potential confounders.

Significance level was set at *p* < 0.05. Statistical analysis was performed with SPSS software version 24.0 (IBM Corp., Armonk, NY, USA).

## 3. Results

Background and baseline characteristics of participant pregnant women and newborns are shown in Table 1. As expected, maternal weight at both 20 weeks and 32 weeks of gestation were higher in pre-gestational overweight and obese mothers compared to normo-weight ones. Overweight and obese women showed lower gestational weight gain than lean ones. Serum fasting glucose at mid pregnancy (24 weeks) and GDM prevalence were also higher in overweight and obese women compared to lean pregnant women. About 40% of women consumed DHA supplements during pregnancy; however, lean women consumed more supplements during the third trimester than obese participants. Obese women had lower education level and also lower social class than lean women; women with university studies used more frequently DHA supplements than women with lower studies (67.2% *n* = 180 vs. 32.8% *n* = 88, *p* ≤ 0.001). Dietary intake of DHA was above the recommendations (>200 mg/d) in the entire cohort and also in the different pre-pregnancy BMI groups. Gestational age at delivery was similar among groups. Regarding the mode of delivery, overweight mothers underwent caesarean section more frequently than the other groups. Babies born to obese mothers presented higher weight and BMI Z-score at birth than newborns from lean and overweight women.

Maternal serum FA profile at 24 weeks of gestation presented several differences between pre-pregnancy BMI groups (Table 2). Among women without GDM, serum DHA percentage was significantly lower in obese mothers compared to lean and overweight volunteers in both unadjusted (*p* = 0.002) and adjusted analysis (*p* = 0.028). In contrast, AA and n-6/n-3 PUFA ratio were higher in obese mothers compared to lean and overweight participants. Despite maternal FA differences, no major differences were observed in venous cord blood by maternal BMI categories. However, higher linoleic acid (18:2 n-6, LA) percentage was observed in arterial cord serum of obese women and tended to have lower arteriovenous LA difference, indicative of lower LA accumulation/retention in fetal tissues (*p* = 0.100) (Table 2).

Associations between serum DHA percentage and maternal/neonatal parameters are shown in Table 3. Higher maternal serum DHA percentage at 24 weeks was associated with higher maternal age, higher dietary DHA intake, and maternal DHA supplementation (β = 0.295, *p* < 0.001), while lower levels were associated with higher pre-pregnancy BMI, previous pregnancies, and maternal smoking during pregnancy. Higher cord venous DHA level was associated with higher maternal DHA status at 24 weeks, DHA supplementation during the third trimester of pregnancy, and gestational age at delivery, while lower levels were associated with higher birthweight. There were also differences by newborn’s sex, although only a non-significant trend towards reduced DHA percentage in cord venous of females was found (Female: 5.38 ± 1.28% vs. male: 5.67 ± 1.28%, *p* = 0.112). Despite maternal serum DHA was positively associated with both umbilical vein and artery levels of DHA, although it was inversely associated with arteriovenous DHA difference and then, with less fetal retention of DHA.

Maternal DHA supplementation in all participants was associated with increased levels of DHA in maternal serum even after adjustment by potential confounders (including pre-pregnancy BMI) in pregnant women without GDM (Table 4). Maternal DHA supplementation in both the first and the second trimester enhanced also maternal DHA serum percentage at 24 weeks if separated into lean and overweight groups. It was not possible to perform logistic regression models in obese group due to the low number of obese women supplemented (13 supplemented vs. 40 non-supplemented). Nevertheless, maternal serum DHA percentage in obese mothers supplemented at the first trimester with DHA was higher than in those non-supplemented (3.62 ± 0.82% vs. 3.08 ± 0.79%, *p* = 0.036), and also in the second trimester (3.58 ± 0.84% vs. 3.07 ± 0.72%, *p* = 0.045). Thus, this confirmed that maternal DHA supplementation was positively associated with maternal serum DHA levels regardless of maternal pre-pregnancy BMI.

Concerning umbilical cord DHA levels, maternal DHA supplementation during pregnancy was also associated with higher DHA percentage in both cord venous and artery in all participants even after adjustment by potential confounders (including pre-pregnancy BMI) (Table 4). The positive association between DHA supplementation and cord serum DHA levels regardless of pre-pregnancy BMI was confirmed using lineal regression analysis by stepwise method; there was a significant positive association between maternal DHA supplementation and DHA in cord venous (*p* < 0.001) but not with pre-pregnancy BMI (*p* = 0.335) or the interaction (DHA supplementation*BMI *p* = 0.853). Similar results were obtained for cord artery DHA (data not shown). However, supplementation with DHA in overweight mothers appears to have an attenuated response compared to lean women since higher DHA enhancement is observed in umbilical cord artery of overweight mothers (overweight OR = 2.58 CI 1.06–6.25, *p* = 0.036 vs. lean OR = 1.56 CI 1.06–2.28, *p* = 0.023). No associations were observed between maternal DHA supplementation and the DHA arteriovenous difference (Table 4).

Regarding the results in women with GDM, maternal serum FA profile in both GDM and healthy women presented similar values for DHA percentage at 24 weeks, although PUFAs were lower in women with GDM mainly due to reduced level of LA (Table 5). Newborns from GDM women tended to have lower DHA percentage in cord venous serum compared to those born from non-GDM women (*p* = 0.061), although this trend did not remain after adjustment for potential confounders (*p* = 0.489) (Table 5). Moreover, higher levels of MUFA were found in cord venous of GDM group as a consequence of higher percentage of oleic acid (18:1 n-9) compared to the non-GDM group. Some minor differences were observed in cord artery FA profile although all of them disappeared after adjustment (Table 5). The arteriovenous difference was similar for all selected FA in both GDM and non-GDM women and hence their offspring tissues FA retention (Table 5).

Maternal DHA supplementation was also associated with higher maternal serum DHA status at 24 weeks in both the total population (adjusted and unadjusted) and in the sub-group of women with GDM (Table 6). However, DHA supplementation only showed positive associations with umbilical cord venous DHA when considering all study participants while there was no association in the GDM sub-group. The huge differences in the number of samples in both cord venous (non-GDM *n* = 319 vs. GDM *n* = 26) and cord artery (non-GDM *n* = 154 vs. GDM *n* = 12) could have masked the GDM-driven differences in this association in the whole population. Linear regression analysis corroborated the association between cord venous DHA and maternal DHA supplementation (*p* < 0.001) while the association of cord venous DHA with GDM in the whole population was non-significant but showed a low *p* tending value (*p* = 0.160) and the interaction was in the same line (DHA supplementation*GDM *p* = 0.296). Similar results were obtained in cord artery (data not shown). Thus, maternal DHA supplementation improved maternal serum DHA in GDM women while there was no benefit for cord DHA status at birth although it cannot be excluded that this lack of association might be due to the low sample size in the GDM sub-group.

## 4. Discussion

In the present study, we found that maternal DHA supplementation in special conditions such as pre-pregnancy obesity and GDM is associated with increased maternal DHA status, while enhancement of offspring DHA status seems to be compromised by maternal GDM condition.

Obese women included in this study presented increased levels of n-6 LC-PUFA while n-3 PUFA, especially DHA, were significantly lower compared to lean pregnant women. These results are in agreement with others [35,36,37]. However, Cinelli et al. reported in a 435 mother-infant pairs cohort that maternal BMI was positively associated with maternal serum DHA [8]. The authors argued that this finding was unlikely related to a higher dietary DHA intake, since most of the pregnant women consumed less than three servings of fish per week, and that this might be due to lower transfer of DHA from mother to fetus [8]. Interestingly, in the present study, the baseline dietary intake of DHA was similar among the three BMI groups and in all cases above the recommendations (>250 mg/d). Nevertheless, obese women tended to consume less DHA supplements, which could be related to their lower DHA percentage in plasma. N6/n-3 PUFA ratio in the maternal serum was also impaired by pre-pregnancy obesity, being significantly higher in obese mothers, which is related to a systemic pro-inflammatory response [38,39]. It is important to note that, in contrast to obese participants, in the present study, maternal serum DHA percentage and n-6/n-3 PUFA ratio were not altered in overweight women. All these evidences together may indicate that an additional n-3 PUFA intake in obese mothers would be desirable in order to promote a healthy FA pattern.

Despite BMI-related alterations in maternal FA plasma profile, no major changes were found neither in venous cord blood, nor in arterial cord blood nor in the arteriovenous FA difference among groups. Only LA was higher in arterial cord blood of obese mothers, while no differences were observed in cord venous blood. This may suggest lower LA fetal retention among obese pregnancy of this FA, which is in line with the altered fetal lipid profile with lower percentage of PUFA, both n-6 and n-3 (including DHA) previously reported by other authors [8]. No other studies are available for arteriovenous difference in obese or overweight women.

Maternal supplementation with 200 mg DHA/day during the first and the second trimester of pregnancy in our study was associated to enhanced maternal serum DHA status regardless of pre-pregnancy BMI. Monthe-Dreze et al. showed an attenuated response to n-3 supplementation in overweight and obese pregnant women resulting in a 50% difference in the effect size compared to lean pregnant women [20]. However, they administered a very high amount of n-3 FA to women (800 mg of DHA + 1200 mg of EPA per day) and 17-hydroxyprogesterone weekly, as a treatment to prevent preterm birth [20]. 17-hydroxyprogesterone might have influenced the results since it has shown DHA biosynthesis promotion in human hepatocytes and liver of ovariectomized rats [40,41]. Other studies in non-pregnant women also described a reduced increase in serum DHA with higher BMI in young women [42] and in women with increased risk of breast cancer [43]. Pregnancy is a special physiological condition in which lipid metabolism is adapted to supply the fetus FA requirements. Our results indicate that the maternal supplementation with DHA was effective in all BMI groups to increase maternal DHA status.

Maternal supplementation with DHA during pregnancy was also associated with increased fetal DHA status, in both cord venous and arterial blood regardless of pre-gestational BMI. This is consistent with previous studies that demonstrated enhanced fetal DHA status after maternal n-3 supplementation in healthy lean pregnant women [44,45,46]. However, regression analysis suggested an attenuated response in overweight women since umbilical artery DHA was especially enhanced in overweight respect to lean women, which may indicate a lower DHA retention in the fetus and hence less effectiveness of dietary DHA supplementation.

Concerning GDM condition, lower percentages of LA and PUFA were found in pregnant women with GDM compared to non-GDM ones, while similar DHA levels were observed for both groups. There are discrepancies in the literature with regard to maternal FA profile in GDM pregnancies; lower, similar, or even higher levels of DHA in GDM have been reported [10,17,47,48,49]. However, newborns of women suffering GDM tended to have lower DHA in cord venous than those from non-GDM women, although these differences disappeared after adjustment by potential confounders. Other authors also reported lower DHA in cord venous despite no changes in maternal blood [10,11].

Maternal DHA supplementation (200 mg/d) was also associated with increased level of DHA in maternal serum in pregnancies complicated with GDM but failed to enhance the newborn DHA status in the GDM sub-group (unadjusted and adjusted analysis). Our results are in line with those reported by Min et al. who showed maternal but not fetal DHA level enhancement in pregnant women with GDM after 600 mg/d DHA supplementation [19]. An impaired materno-fetal transfer of DHA has been demonstrated in GDM pregnancies [12,50,51], which can be explained at least in part by some alterations in FA transport protein associated to phospholipid transfer such as the major facilitator superfamily domain-containing 2a and FA transport protein number 4 [15,16]. In vitro experiments with human choriocarcinoma BeWo cells have shown a regulatory role of insulin on some specific FA carriers that may be related to the higher adiposity observed in GDM neonates [52]. Ortega-Senovilla et al. suggested that lower cord DHA could be due to enhanced DHA utilization by fetal tissues (e.g., active conversion or metabolism) in GDM rather than unpaired transport across the placenta [17,51]. They obtained lower DHA percentage in arterial cord blood of GDM while similar DHA content in venous cord blood respect to healthy controls [17]. A similar observation has been made in neonates from mothers with type I diabetes [18]. We cannot confirm a higher fetal uptake of DHA by the fetus from GDM mothers since there were no differences for DHA in the arteriovenous difference between GDM and non-GDM subjects, and the number of patients in the present study was much higher than in previous ones. A disturbed placental transfer in GDM seems to be the main reason for lower cord DHA in the offspring of GDM according to the present results.

The strengths of the present study include the quantification of FA in both venous and arterial cord serum samples in a population-based prospective birth cohort, which allows us to estimate for the first time not only the associations between the maternal DHA supplementation during pregnancy and fetal DHA levels but also the fetal tissue retention of DHA. Our study also presents some limitations. The small number of women included in the supplemented obese group that limited the statistical power of some analyses in this group. In this line, we cannot exclude that the lack of association between maternal DHA supplementation and cord venous DHA percentage in GDM group might be due to the low number of subjects included in this sub-group, although the results were consistent among the three trimesters by DHA supplementation. Information about DHA supplementation was obtained from self-reports of participant pregnant women, which could have also biased the results obtained. Finally, the influence of other residual confounding factors not included in the present statistical analyses, bias due to missing data, in the measurement of outcomes or perhaps also in selection of the reported results cannot be excluded.

## 5. Conclusions

In conclusion, maternal DHA supplementation during pregnancy was associated with both maternal and newborn increased DHA status regardless of the pre-gestational BMI of the mother, although supplementation with DHA at third trimester in overweight women was associated with higher levels of arterial DHA cord serum and less DHA fetal retention. However, in women with GDM, DHA supplementation was associated with increased maternal but not cord venous DHA status. Our results support an altered materno-fetal transfer of DHA during pregnancy instead of higher DHA fetal accumulation since no changes were found in arteriovenous DHA difference in GDM women. A higher consumption of n-3 LC-PUFA might be desirable in obese women in order to improve maternal serum FA n6/n3 ratio. In addition, neonates born from GDM women may need DHA fortification after birth, especially those non breast-fed.

## Figures and Tables

**Table 1:** Sociodemographic characteristics of participants categorized by maternal pre-pregnancy body mass index (BMI). The Nutrition in Early Life and Asthma (NELA) study (2015–2018).

	All	Maternal Pre-Pregnancy BMI									*p*
		Lean			Overweight			Obese			
	*n* = 627	(18.5–24.9 kg/m^2^) *n* = 429			(25–29.9 kg/m^2^) *n* = 143			(≥30 kg/m^2^) *n* = 55			
**Mothers**											
Age (years)	33.23 ± 4.44	33.10	±	4.36	33.55	±	4.41	33.43	±	5.13	0.536
Height (cm)	164.00 ± 5.79	164.04	±	5.79	163.35	±	5.71	165.44	±	5.80	0.073
Maternal weight (kg)											
Pregestational	65.09 ± 12.68	58.96	±	5.98 ^a^	71.93	±	6.32 ^b^	95.08	±	11.73 ^c^	<0.001
20th weeks	71.51 ± 12.33	65.87	±	7.00 ^a^	77.68	±	6.74 ^b^	98.78	±	11.56 ^c^	0.001
32nd weeks	77.64 ± 12.41	72.33	±	7.57 ^a^	83.76	±	7.46 ^b^	104.00	±	13.02 ^c^	0.001
Pregestational BMI (kg/m^2^)	24.16 ± 4.30	21.89	±	1.68 ^a^	26.92	±	1.39 ^b^	34.69	±	3.54 ^c^	<0.001
Gestational weight gain (kg)	12.17 ± 4.96	12.95	±	4.19 ^a^	11.32		4.75 ^b^	8.23	±	8.04 ^c^	<0.001
Educational level											<0.001
Incomplete secondary or less	117 (18.66%)	78 (18.18%) ^a^			24 (16.78%) ^ab^			15 (27.27%) ^b^			
Complete secondary	160 (25.52%)	87 (20.28%) ^a^			54 (37.76%) ^b^			19 (34.55%) ^b^			
University	350 (55.82%)	264 (61.54%) ^a^			65 (45.45%) ^b^			21 (38.18%) ^b^			
Social class											0.006
Unemployed	127 (20.26%)	78 (18.18%) ^a^			33 (23.08%) ^ab^			16 (29.09%) ^b^			
Semiskilled/unskilled	121 (19.30%)	74 (17.25%) ^a^			31 (21.68%) ^ab^			16 (29.09%) ^b^			
Skilled	148 (23.60%)	99 (23.08%) ^a^			35 (24.48%) ^a^			14 (25.46%) ^a^			
Managers/technicians	231 (36.84%)	178 (41.49%) ^a^			44 (30.77%) ^b^			9 (16.36%) ^c^			
Smoking during pregnancy (yes)	99 (15.79%)	63 (14.69%)			23 (16.08%)			13 (23.64%)			0.229
Alcohol during pregnancy (yes)	33 (5.26%)	20 (4.66%)			11 (7.69%)			2 (3.64%)			0.317
Dietary DHA intake (mg/d)	270.16 ± 196.53	275.24	±	198.30	259.33	±	174.06	259.42	±	234.49	0.661
Use of DHA supplements (≥200 mg/d)											
1st trimester	246 (39.23%)	179 (41.72%)			52 (36.36%)			15 (27.27%)			0.086
2nd trimester	254 (40.51%)	185 (43.12%)			53 (37.06%)			16 (29.09%)			0.086
3rd trimester	265 (42.26%)	196 (45.69%) ^a^			54 (37.76%) ^ab^			15 (27.27%) ^b^			0.016
GDM (yes)	49 (7.81%)	19 (4.43%) ^a^			22 (15.38%) ^b^			8 (14.55%) ^b^			<0.001
Serum glucose 24 weeks (mg/dl)	77.18 ± 7.07	76.15	±	6.42 ^a^	78.85	±	7.84 ^b^	81.48	±	7.87 ^b^	<0.001
Pre-eclampsia (yes)	6 (0.96%)	3 (0.78%)			3 (2.36%)			0			0.250
Parity											0.446
0	316 (50.40%)	221 (51.52%)			73 (51.05%)			22 (40.00%)			
1	244 (38.92%)	161 (37.53%)			58 (40.56%)			25 (45.45%)			
>1	67 (10.69%)	47 (10.96%)			12 (8.39%)			8 (14.55%)			
Time from last pregnancy											0.340
<12 months	74 (11.80%)	51 (11.89%)			19 (13.29%)			4 (7.27%)			
>12 months	296 (47.21%)	196 (45.49)			67 (46.85%)			33 (60.00%)			
Mode of delivery											0.014
Vaginal	491 (78.31%)	347 (81.84%) ^a^			101 (71.13%) ^b^			40 (72.73%) ^ab^			
Caesarean section	133 (21.21%)	77 (18.16%) ^a^			41 (28.87%) ^b^			15 (27.27%) ^ab^			
**Newborns**											
Gestational age (weeks)	39.66 ± 1.44	39.62	±	1.40	39.67	±	1.62	39.98	±	1.23	0.212
Weight (kg)	3.26 ± 0.46	3.24	±	0.44 ^a^	3.24	±	0.50 ^a^	3.48	±	0.39 ^b^	0.001
Height (cm)	50.68 ± 2.22	50.63	±	2.15	50.56	±	2.58	51.33	±	1.72	0.071
BMI Z-score	−0.29 ± 1.05	−0.33	±	1.02 ^a^	−0.38	±	1.13 ^a^	0.26	±	0.96 ^b^	0.001
Nutritional status											
Low birth weight	27 (4.31%)	16 (4.42 %)			9 (7.56 %)			2 (4.08 %)			0.380
Normal weight	446 (71.13%)	311 (85.91 %)			98 (82.35 %)			37 (75.51 %)			0.189
Overweight risk	47 (7.50%)	29 (8.01 %)			10 (8.40 %)			8 (16.33 %)			0.161
Overweight	9 (1.44%)	6 (1.66 %)			1 (0.84 %)			2 (4.08 %)			0.383
Obese	1 (0.16%)	0			1 (0.84 %)			0			0.163
Head circumference (cm)	34.38 ± 1.56	34.31	±	1.54	34.43	±	1.67	34.84	±	1.39	0.058
Sex											0.149
Male	314 (50.08%)	226 (52.80%)			64 (44.76%)			24 (43.64%)			
Female	312 (49.76%)	202 (47.20%)			79 (55.24%)			31 (56.36%)			

Data are mean ± SD or *n* (%). *p*-values are ANOVA, Kruskal–Wallis, or Chi square test. BMI: Body mass index. Values not sharing the same superscript letter indicate significant differences between BMI categories (*p* < 0.05).

**Table 2:** Maternal and newborn fatty acid profile according to pre-pregnancy body mass index (BMI). The NELA study (2015–2018).

	Maternal Pre-Pregnancy BMI										
	Lean			Overweight			Obese			*p* *	*p* ^†^
	(18.5–24.9 kg/m^2^)			(25–29.9 kg/m^2^)			(≥30 kg/m^2^)				
	g/100 g Fatty Acids										
**Materal Serum (24 Weeks)**	***n*** **= 397**			***n*** **= 116**			***n*** **= 47**				
16:0	23.40	±	1.83 ^a^	23.57	±	1.51 ^a^	24.30	±	1.61 ^b^	0.004	0.016
18:0	6.40	±	1.26 ^a^	6.06	±	1.07 ^b^	6.29	±	1.07 ^ab^	0.026	0.034
18:1 n9	18.62	±	2.38	18.49	±	2.43	18.20	±	2.20	0.496	0.527
18:2 n6	30.09	±	3.22	29.96	±	3.53	29.84	±	3.01	0.840	0.782
18:3 n3	0.34	±	0.18 ^a^	0.33	±	0.18 ^ab^	0.26	±	0.14 ^b^	0.036	0.058
20:4 n6	6.76	±	1.25 ^a^	7.09	±	1.22 ^b^	7.12	±	1.40 ^ab^	0.017	0.015
20:5 n3	0.44	±	0.40	0.43	±	0.32	0.34	±	0.30	0.238	0.369
22:6 n3 (DHA)	3.65	±	0.88 ^a^	3.66	±	0.75 ^a^	3.20	±	0.77 ^b^	0.002	0.028
SFA	32.81	±	2.70	32.48	±	1.89	33.43	±	1.82	0.085	0.198
MUFA	22.80	±	2.64	22.84	±	2.85	22.52	±	2.42	0.765	0.819
PUFA	44.38	±	2.99	44.68	±	3.11	44.05	±	2.96	0.444	0.448
Ratio PUFA n6/n3	9.28	±	2.86 ^a^	9.28	±	2.50 ^a^	10.92	±	3.34 ^b^	0.001	0.012
LC-PUFA n3	4.30	±	1.27 ^a^	4.26	±	1.05 ^a^	3.69	±	1.01 ^b^	0.005	0.060
LC-PUFA n6	9.39	±	1.49 ^a^	9.83	±	1.44 ^b^	9.97	±	1.44 ^b^	0.002	0.003
**Venous cord blood**	*n* = 218			*n* = 67			*n* = 25				
16:0	26.91	±	1.73	26.95	±	1.39	27.19	±	1.22	0.723	0.640
18:0	10.93	±	2.21	10.74	±	2.02	10.78	±	1.68	0.802	0.897
18:1 n9	15.74	±	2.39	16.04	±	1.73	15.74	±	1.61	0.607	0.685
18:2 n6	12.02	±	2.77	12.27	±	2.99	11.63	±	1.46	0.595	0.834
18:3 n3	0.02	±	0.08	0.04	±	0.12	0.02	±	0.05	0.427	0.559
20:4 n6	12.68	±	2.00	12.67	±	1.92	12.91	±	1.47	0.850	0.997
20:5 n3	0.37	±	0.73	0.39	±	0.38	0.45	±	0.58	0.837	0.844
22:6 n3 (DHA)	5.57	±	1.69	5.56	±	1.31	5.13	±	1.23	0.417	0.914
SFA	41.45	±	3.19	41.12	±	2.84	41.74	±	2.83	0.641	0.679
MUFA	22.87	±	3.05	23.01	±	2.02	22.95	±	2.24	0.934	0.955
PUFA	35.67	±	3.44	35.81	±	2.42	35.30	±	2.01	0.790	0.865
Ratio n6/n3	5.42	±	3.68	5.12	±	1.45	5.54	±	1.70	0.760	0.593
LC-PUFA n3	6.04	±	2.18	6.04	±	1.55	5.67	±	1.41	0.678	0.985
LC-PUFA n6	16.96	±	2.41	16.85	±	2.11	17.33	±	1.84	0.681	0.980
**Arterial cord blood**	*n* = 103			*n* = 32			*n* = 12				
16:0	27.64	±	2.30	27.96	±	1.08	27.13	±	1.77	0.478	0.546
18:0	10.63	±	1.35 ^ab^	10.83	±	1.09 ^a^	9.79	±	0.96 ^b^	0.052	0.032
18:1 n9	15.27	±	1.91	15.48	±	1.33	16.02	±	1.64	0.368	0.134
18:2 n6	11.55	±	1.96 ^a^	11.49	±	1.71 ^ab^	12.78	±	2.29 ^b^	0.107	0.047
18:3 n3	0.00	±	0.03	0.01	±	0.04	0.01	±	0.05	0.544	0.580
20:4 n6	12.98	±	1.82	12.65	±	1.46	12.13	±	0.95	0.210	0.215
20:5 n3	0.19	±	0.37	0.13	±	0.31	0.05	±	0.12	0.293	0.729
22:6 n3 (DHA)	5.48	±	1.33	5.63	±	1.22	5.26	±	1.44	0.707	0.951
SFA	42.76	±	4.71	42.49	±	1.92	40.76	±	2.53	0.287	0.112
MUFA	22.42	±	2.68	22.80	±	1.87	23.76	±	2.34	0.194	0.070
PUFA	34.82	±	3.44	34.71	±	1.90	35.47	±	2.23	0.755	0.568
Ratio n6/n3	5.24	±	1.21	5.17	±	1.45	5.99	±	2.04	0.166	0.683
LC-PUFA n3	5.74	±	1.52	5.85	±	1.35	5.40	±	1.49	0.668	0.978
LC-PUFA n6	17.01	±	2.05	16.83	±	1.58	16.70	±	1.22	0.808	0.569
**Arteriovenous difference**	*n* = 95			*n* = 31			*n* = 12				
16:0	−0.79	±	2.61	−1.08	±	1.50	0.20	±	1.61	0.273	0.214
18:0	0.49	±	2.46	−0.08	±	1.51	1.22	±	1.69	0.204	0.304
18:1 n9	0.44	±	2.54	0.73	±	1.74	−0.12	±	1.11	0.550	0.210
18:2 n6	0.53	±	2.24	0.64	±	1.72	−0.92	±	1.71	0.066	0.100
18:3 n3	0.02	±	0.09	0.03	±	0.14	0.01	±	0.09	0.742	0.836
20:4 n6	−0.39	±	2.21	−0.19	±	1.23	−0.32	±	1.20	0.882	0.827
20:5 n3	0.16	±	0.63	0.21	±	0.36	0.47	±	0.78	0.254	0.294
22:6 n3 (DHA)	0.21	±	1.75	−0.02	±	0.61	−0.44	±	0.63	0.329	0.598
SFA	−1.28	±	5.57	−1.51	±	2.77	1.56	±	3.18	0.148	0.060
MUFA	0.30	±	3.38	0.52	±	1.68	−0.50	±	1.57	0.592	0.173
PUFA	0.96	±	4.39	0.87	±	1.42	−1.06	±	1.99	0.215	0.302
Ratio n6/n3	−0.05	±	1.45	−0.04	±	0.66	−0.35	±	0.68	0.735	0.641
LC-PUFA n3	0.42	±	1.99	0.18	±	0.88	0.05	±	0.54	0.943	0.913
LC-PUFA n6	−0.10	±	2.82	−0.08	±	1.68	−0.35	±	1.78	0.675	0.897

Data are mean ± SD. * Unadjusted analysis of variance (ANOVA). ^†^ Analysis of covariance (ANCOVA) test adjusted by the following potential confounders: Maternal age, education level, smoking, dietary DHA intake, DHA supplementation, and previous pregnancies in the case of maternal parameters; analysis adjusted by maternal serum DHA (24 weeks), DHA supplementation in the third trimester, gestational age, sex, and birth weight for newborn parameters. BMI: Body mass index; LC-PUFA: Long-chain polyunsaturated fatty acids (≥2 double bounds and >18 carbons); MUFA: Monounsaturated fatty acids; PUFA: Polyunsaturated fatty acids (≥2 double bounds); SFA: Saturated fatty acids. Values not sharing the same superscript letter indicate significant differences between BMI categories (*p* < 0.05).

**Table 3:** Associations between serum docosahexaenoic acid (DHA) percentage and maternal/neonatal variables. The NELA study (2015–2018).

	B	β	CI (95%)	*p*
**Maternal serum DHA 24 weeks (*n* = 526)**				<0.001
Maternal age	0.029	0.147	(0.012, 0.045)	0.001
Educational level	0.074	0.079	(−0.004, 0.152)	0.064
Smoking	−0.233	−0.097	(−0.421, −0.046)	0.015
Previous pregnancies	−0.286	−0.222	(−0.394, −0.178)	<0.001
Pre-pregnancy BMI	−0.016	−0.080	(−0.031, −0.001)	0.042
Dietary DHA	0.495	0.115	(0.163, 0.827)	0.004
DHA supplementation 1st trimester	0.524	0.295	(0.387, 0.661)	<0.001
**Cord venous DHA (*n* = 299)**				<0.001
Maternal serum DHA 24 weeks	0.308	0.170	(0.093, 0.522)	0.005
DHA supplementation 3er trimester	0.467	0.143	(0.080, 0.854)	0.018
Gestational age	0.282	0.226	(0.127, 0.436)	<0.001
Sex	−0.363	−0.114	(−0.714, −0.012)	0.043
Birth weight	0.000	−0.128	(−0.001, 0.000)	0.045
**Cord artery DHA (*n* = 143)**				<0.001
Maternal serum DHA 24 weeks	0.645	0.422	(0.403, 0.887)	<0.001
DHA supplementation 3er trimester	0.088	0.033	(−0.335, 0.510)	0.681
Gestational age	0.285	0.293	(0.132, 0.438)	<0.001
Sex	0.293	0.111	(−0.087, 0.673)	0.130
Birth weight	0.000	−0.098	(−0.001, 0.000)	0.216
**Arteriovenous difference (*n* = 135)**				0.008
Maternal serum DHA 24 weeks	−0.422	−0.238	(−0.750, −0.094)	0.012
DHA supplementation 3er trimester	0.692	0.227	(0.123, 1.262)	0.018
Gestational age	−0.083	−0.075	(−0.289, 0.123)	0.428
Sex	−0.564	−0.186	(−1.076, −0.052)	0.031
Birth weight	0.001	−0.068	(−0.001, 0.000)	0.470

Associations were evaluated using lineal regression analyses. β and *p* are corrected values after adjustment for potential confounders: Maternal age, education level, smoking, dietary DHA intake, DHA supplementation, previous pregnancies, and pre-pregnancy BMI in the case of maternal DHA; analysis adjusted by maternal serum DHA (24 weeks), DHA supplementation in the third trimester, gestational age, sex, and birth weight for cord blood DHA. BMI: Body mass index. CI: Confidence interval. Significance level set at *p* < 0.05.

**Table 4:** Logistic regression analysis assessing the odds of having different response to dietary DHA supplements depending on maternal pre-pregnancy body mass index (BMI). The NELA study (2015–2018).

	All				Maternal Pre-Pregnancy BMI							
					Lean (18.5–24.9 kg/m^2^)				Overweight (25–29.9 kg/m^2^)			
	Unadjusted		Adjusted ^†^		Unadjusted		Adjusted *		Unadjusted		Adjusted *	
	OR (95% CI)	*p*	OR (95% CI)	*p*	OR (95% CI)	*p*	OR (95% CI)	*p*	OR (95% CI)	*p*	OR (95% CI)	*p*
**Maternal serum DHA 24 wk**	Suppl. yes/no *n* = 225/361		Suppl. yes/no *n* = 205/341		Suppl. yes/no *n* = 162/233		Suppl. yes/no *n* = 150/221		Suppl. yes/no *n* = 42/74		Suppl. yes/no *n* = 38/71	
DHA supl. 1st trimester	2.68 (1.09–3.43)	<0.001	2.50 (1.90–3.29)	<0.001	2.86 (2.10–3.88)	<0.001	2.73 (1.94–3.84)	<0.001	2.60 (1.44–4.69)	0.001	1.89 (0.97–3.65)	0.06
	Suppl. yes/no *n* = 233/353		Suppl. yes/no *n* = 213/333		Suppl. yes/no *n* = 169/228		Suppl. yes/no *n* = 156/215		Suppl. yes/no *n* = 43/63		Suppl. yes/no *n* = 39/70	
DHA supl. 2nd trimester	2.40 (1.89–3.04)	<0.001	2.26 (1.74–2.95)	<0.001	2.45 (1.83–3.27)	<0.001	2.31 (1.67–3.19)	<0.001	2.88 (1.57–5.28)	0.001	2.21 (1.14–4.30)	0.019
**Cord venous DHA**	Suppl. yes/no *n* = 112/207		Suppl. yes/no *n* = 110/207		Suppl. yes/no *n* = 81/137		Suppl. yes/no *n* = 81/137		Suppl. yes/no *n* = 23/44		Suppl. yes/no *n* = 23/44	
DHA supl. 1st trimester	1.32 (1.11–1.57)	0.002	1.35 (1.12–1.62)	0.001	1.27 (0.04–1.55)	0.017	1.32 (1.07–1.63)	0.01	1.42 (0.95–2.11)	0.084	1.53 (0.98–2.40)	0.062
	Suppl. yes/no *n* = 119/200		Suppl. yes/no *n* = 118/199		Suppl. yes/no *n* = 86/132		Suppl. yes/no *n* = 86/132		Suppl. yes/no *n* = 25/42		Suppl. yes/no *n* = 25/42	
DHA supl. 2nd trimester	1.36 (1.14–1.63)	0.001	1.39 (1.15–1.68)	0.001	1.36 (1.10–1.68)	0.005	1.42 (1.13–1.78)	0.003	1.46 (0.98–2.18)	0.062	1.54 (0.98–2.40)	0.06
	Suppl. yes/no *n* = 128/191		Suppl. yes/no *n* = 126/191		Suppl. yes/no *n* = 92/126		Suppl. yes/no *n* = 92/126		Suppl. yes/no *n* = 26/41		Suppl. yes/no *n* = 26/41	
DHA supl. 3rd trimester	1.36 (1.14–1.62)	0.001	1.39 (1.15–1.68)	0.001	1.30 (1.06–1.59)	0.011	1.37 (1.09–1.71)	0.006	1.49 (1.00–2.22)	0.053	1.59 (1.01–2.50)	0.047
**Cord artery DHA**	Suppl. yes/no *n* = 55/99		Suppl. yes/no *n* = 53/99		Suppl. yes/no *n* = 39/64		Suppl. yes/no *n* = 39/64		Suppl. yes/no *n* = 11/21		Suppl. yes/no *n* = 11/21	
DHA supl. 1st trimester	1.37 (1.04–1.80)	0.023	1.35 (1.00–0.82)	0.052	1.28 (0.93–1.76)	0.138	1.24 (0.87–1.79)	0.236	1.65 (0.86–3.18)	0.135	2.02 (0.84–4.87)	0.116
	Suppl. yes/no *n* = 59/95		Suppl. yes/no *n* = 58/94		Suppl. yes/no *n* = 41/62		Suppl. yes/no *n* = 41/62		Suppl. yes/no *n* = 13/19		Suppl. yes/no *n* = 13/19	
DHA supl. 2nd trimester	1.41 (1.07–1.85)	0.014	1.36 (1.01–1.83)	0.04	1.29 (0.94–1.78)	0.118	1.36 (0.95–1.94)	0.097	1.92 (0.98–3.77)	0.058	2.24 (0.94–5.33)	0.067
	Suppl. yes/no *n* = 66/88		Suppl. yes/no *n* = 64/88		Suppl. yes/no *n* = 46/57		Suppl. yes/no *n* = 46/57		Suppl. yes/no *n* = 14/18		Suppl. yes/no *n* = 14/18	
DHA supl. 3rd trimester	1.55 (1.17–2.05)	0.002	1.52 (1.20–2.06)	0.007	1.46 (1.04–2.05)	0.028	1.56 (1.06–2.28)	0.023	2.16 (1.06–4.37)	0.033	2.58 (1.06–6.25)	0.036
**Arteriovenous difference**	Suppl. yes/no *n* = 51/93		Suppl. yes/no *n* = 49/93		Suppl. yes/no *n* = 35/60		Suppl. yes/no *n* = 35/60		Suppl. yes/no *n* = 11/20		Suppl. yes/no *n* = 11/20	
DHA supl. 1st trimester	1.14 (0.89–1.46)	0.305	1.15 (0.89–1.49)	0.275	1.08 (0.85–1.37)	0.527	1.12 (0.87–1.44)	0.38	2.30 (0.59–8.92)	0.228	2.49 (0.50–12.49)	0.269
	Suppl. yes/no *n* = 54/89		Suppl. yes/no *n* = 54/88		Suppl. yes/no *n* = 37/58		Suppl. yes/no *n* = 37/58		Suppl. yes/no *n* = 13/18		Suppl. yes/no *n* = 13/18	
DHA supl. 2nd trimester	1.15 (0.89–1.50)	0.278	1.19 (0.90–1.57)	0.215	1.15 (0.88–1.51)	0.304	1.18 (0.89–1.56)	0.252	1.58 (0.46–5.41)	0.469	1.37 (0.30–6.27)	0.682
	Suppl. yes/no *n* = 62/82		Suppl. yes/no *n* = 60/82		Suppl. yes/no *n* = 42/53		Suppl. yes/no *n* = 42/53		Suppl. yes/no *n* = 14/17		Suppl. yes/no *n* = 14/17	
DHA supl. 3rd trimester	1.16 (0.89–1.53)	0.274	1.19 (0.90–1.57)	0.225	1.14 (0.87–1.49)	0.355	1.18 (0.89–1.57)	0.25	1.22 (0.37–4.01)	0.741	1.04 (0.24–4.50)	0.956

* Analyses adjusted for potential confounders: Maternal age, education level, smoking, dietary DHA intake, and previous pregnancies in the case of maternal DHA; previous pregnancies, gestational age, sex, and birth weight for cord venous, cord artery, and arteriovenous DHA difference. ^†^ Analysis adjusted by the potential confounders listed before plus maternal pre-pregnancy BMI. BMI: Body mass index. CI: Confidence interval; OR: Odds ratio. Significance level set at *p* < 0.05.

**Table 5:** Maternal and newborn fatty acid profile in healthy and gestational diabetes mellitus (GDM) pregnancies. The NELA study (2015–2018).

	Non-GDM			GDM			*p* *	*p* ^†^
	g/100 g Fatty Acids							
**Maternal Serum (24 Weeks)**	***n*** **= 586**			***n*** **= 49**				
16:0	23.53	±	1.76	24.38	±	2.34	0.017	0.014
18:0	6.31	±	1.20	6.18	±	0.93	0.448	0.458
18:1 n9	18.52	±	2.38	19.08	±	2.84	0.121	0.223
18:2 n6	30.03	±	3.29	28.19	±	4.05	<0.001	0.001
18:3 n3	0.33	±	0.18	0.34	±	0.12	0.721	0.784
20:4 n6	6.87	±	1.26	7.11	±	1.27	0.194	0.094
20:5 n3	0.43	±	0.38	0.40	±	0.31	0.582	0.588
22:6 n3 (DHA)	3.62	±	0.86	3.58	±	0.83	0.782	0.536
SFA	32.81	±	2.46	33.40	±	2.66	0.108	0.216
MUFA	22.76	±	2.68	23.57	±	3.05	0.044	0.083
PUFA	44.42	±	3.04	43.02	±	3.90	0.017	0.013
Ratio PUFA n6/n3	9.41	±	2.87	9.06	±	2.22	0.408	0.548
LC-PUFA n3	4.24	±	1.21	4.14	±	1.10	0.570	0.448
LC-PUFA n6	9.55	±	1.49	9.99	±	1.48	0.048	0.017
**Venous cord blood**	*n* = 319			*n* = 26				
16:0	26.93	±	1.65	26.43	±	2.10	0.150	0.281
18:0	10.88	±	2.16	10.34	±	1.78	0.217	0.148
18:1 n9	15.79	±	2.20	17.00	±	2.38	0.008	0.004
18:2 n6	12.09	±	2.78	11.60	±	3.71	0.405	0.400
18:3 n3	0.03	±	0.10	0.08	±	0.14	0.068	0.036
20:4 n6	12.70	±	1.93	12.85	±	2.53	0.697	0.865
20:5 n3	0.37	±	0.65	0.28	±	0.27	0.471	0.504
22:6 n3 (DHA)	5.53	±	1.58	5.14	±	0.92	0.061	0.489
SFA	41.39	±	3.15	40.87	±	3.04	0.421	0.395
MUFA	22.89	±	2.78	24.47	±	2.90	0.006	0.004
PUFA	35.70	±	3.17	34.64	±	3.14	0.100	0.076
Ratio PUFA n6/n3	5.37	±	3.16	5.24	±	1.51	0.836	0.619
LC-PUFA n3	6.01	±	1.99	5.62	±	1.02	0.333	0.645
LC-PUFA n6	16.96	±	2.29	16.71	±	2.69	0.605	0.281
**Arterial cord blood**	*n* = 154			*n* = 12				
16:0	27.69	±	2.04	27.90	±	0.87	0.720	0.655
18:0	10.62	±	1.27	11.22	±	1.15	0.112	0.213
18:1 n9	15.34	±	1.76	15.22	±	2.14	0.821	0.601
18:2 n6	11.67	±	1.98	10.43	±	1.27	0.035	0.187
18:3 n3	0.01	±	0.03	n.d.			-	-
20:4 n6	12.85	±	1.68	14.41	±	2.66	0.004	0.089
20:5 n3	0.16	±	0.34	0.06	±	0.11	0.021	0.874
22:6 n3 (DHA)	5.47	±	1.31	5.12	±	0.70	0.365	0.601
SFA	42.56	±	4.06	42.92	±	2.14	0.762	0.787
MUFA	22.58	±	2.49	22.39	±	2.88	0.799	0.789
PUFA	34.86	±	3.07	34.69	±	2.43	0.854	0.900
Ratio PUFA n6/n3	5.33	±	1.36	5.69	±	1.01	0.370	0.371
LC-PUFA n3	5.70	±	1.47	5.23	±	0.77	0.077	0.652
LC-PUFA n6	16.96	±	1.88	18.48	±	2.67	0.010	0.186
**Arteriovenous difference**	*n* = 144			*n* = 9				
16:0	−0.75	±	2.30	−2.05	±	2.19	0.102	0.187
18:0	0.46	±	2.26	−0.50	±	0.76	0.210	0.121
18:1 n9	0.45	±	2.24	1.34	±	1.94	0.242	0.439
18:2 n6	0.42	±	2.07	1.09	±	1.66	0.341	0.724
20:4 n6	−0.36	±	1.92	−0.57	±	1.24	0.743	0.833
20:5 n3	0.20	±	0.59	0.10	±	0.26	0.625	0.600
22:6 n3 (DHA)	0.10	±	1.48	0.07	±	0.31	0.957	0.732
SFA	−1.02	±	4.87	−1.89	±	2.12	0.598	0.898
MUFA	0.26	±	2.90	1.16	±	1.85	0.360	0.558
PUFA	0.73	±	3.72	0.73	±	1.63	0.999	0.798
Ratio PUFA n6/n3	−0.08	±	1.25	−0.08	±	0.39	0.998	0.883
LC-PUFA n3	0.33	±	1.68	0.15	±	0.29	0.742	0.555
LC-PUFA n6	−0.14	±	2.48	−0.53	±	1.71	0.646	0.908

Data are mean ± SD. * Unpaired Student t-test. ^†^ Analysis of covariance (ANCOVA) test adjusted by the following potential confounders: maternal age, education level, smoking, dietary DHA intake, DHA supplementation and previous pregnancies in the case of maternal fatty acids; analysis adjusted by maternal serum DHA (24 weeks), DHA supplementation in the third trimester, gestational age, sex, and birth weight for newborn parameters. GDM: Gestational diabetes mellitus; LC-PUFA: Long-chain polyunsaturated fatty acids (≥2 double bounds and >18 carbons); MUFA: Monounsaturated fatty acids; n.d.: Non detected. PUFA: Polyunsaturated fatty acids (≥2 double bounds); SFA: Saturated fatty acids. Significance level set at *p* < 0.05.

**Table 6:** Logistic regression analysis assessing the odds of having different response to dietary DHA supplements in pregnancies affected by gestational diabetes mellitus. The NELA study (2015–2018).

	All				GDM			
	Unadjusted		Adjusted ^†^		Unadjusted		Adjusted *	
	OR (95% CI)	*p*	OR (95% CI)	*p*	OR (95% CI)	*p*	OR (95% CI)	*p*
**Maternal Serum DHA 24 wk**	**Suppl. Yes/No *n* = 250/385**		**Suppl. Yes/No *n* = 228/363**		**Suppl. Yes/No *n* = 25/24**		**Suppl. Yes/No *n* = 22/22**	
DHA supl. 1st trimester	2.75 (2.17–3.49)	<0.001	2.58 (1.98–3.36)	<0.001	4.53 (1.66–12.37)	0.003	4.07 (1.29–12.78)	0.016
	Suppl. yes/no *n* = 258/377		Suppl. yes/no *n* = 236/355		Suppl. yes/no *n* = 25/24		Suppl. yes/no *n* = 23/21	
DHA supl. 2nd trimester	2.57 (2.04–3.24)	<0.001	2.40 (1.85–3.10)	<0.001	9.95 (2.59–38.19)	0.001	9.84 (2.15–45.00)	0.003
**Cord venous DHA**	Suppl. yes/no *n* = 124/221		Suppl. yes/no *n* = 124/221		Suppl. yes/no *n* = 12/14		Suppl. yes/no *n* = 12/14	
DHA supl. 1st trimester	1.33 (1.12–1.57)	0.001	1.35 (1.13–1.62)	0.001	2.01 (0.73–5.56)	0.177	1.34 (0.47–3.79)	0.584
	Suppl. yes/no *n* = 132/213		Suppl. yes/no *n* = 132/213		Suppl. yes/no *n* = 13/13		Suppl. yes/no *n* = 13/13	
DHA supl. 2nd trimester	1.34 (1.13–1.60)	0.001	1.36 (1.14–1.63)	0.001	1.29 (0.54–3.08)	0.570	0.86 (0.33–2.25)	0.757
	Suppl. yes/no *n* = 140/205		Suppl. yes/no *n* = 140/205		Suppl. yes/no *n* = 12/14		Suppl. yes/no *n* = 12/14	
DHA supl. 3rd trimester	1.37 (1.15–1.6.)	<0.001	1.40 (1.16–1.68)	<0.001	2.33 (0.79–6.86)	0.125	1.68 (0.52–5.45)	0.385

* Analyses adjusted for potential confounders: maternal age, education level, smoking, dietary DHA intake, and previous pregnancies in the case of maternal DHA; previous pregnancies, gestational age, sex, and birth weight for cord venous DHA. ^†^ Analysis adjusted by the potential confounders listed before plus GDM condition. CI: Confidence interval; OR, Odds ratio. Significance level set at *p* < 0.05.

## Data Availability

The data presented in this study are available on request from the corresponding author.

## Supplementary Materials

The following are available online at https://www.mdpi.com/2072-6643/13/3/843/s1, Figure S1: Directed acyclic graph (DAG) used to explore potential variables (box) that are related to maternal (A) and neonatal serum DHA (B). BMI, body mass index; GDM, gestational diabetes mellitus.

## NELA Study Group

ME Candel-Torralba ^1^, L Garcia-Marcos (PI) ^1,2,4,18^, MJ Gimenez-Banon ^1^, A Martinez-Torres ^1,2,18^, E Morales (PI) ^1,3^, V Perez-Fernandez ^1,4,18^, M Sanchez-Solis ^1,2,4,18^, A Nieto ^1,5^, MT Prieto-Sanchez ^1,5^, M Sanchez-Ferrer ^1,5^, L Fernanez-Palacios ^1,6^, VP Gomez-Gomez ^1,6^, C Martinez-Gracia ^1,6^, P Peso-Echarri ^1,6^, G Ros-Berruezo ^1,6^, M Santaella-Pascual ^1,6^, A Gazquez ^1,7^, E Larque ^1,7^, MT Pastor-Fajardo ^1,7^, M Sanchez-Campillo ^1,7^, A Serrano-Munuera ^1,7^, M Zornoza-Moreno ^1,7^, P Jimenez-Guerrero ^1,8^, E Adomnei ^1,9^, JJ Arense-Gonzalo ^1,9^, J Mendiola ^1,9^, F Navarro-Lafuente ^1,9^, AM Torres-Cantero ^1,9^, C Salvador-Garcia ^10^, M Segovia-Hernandez ^1,11^, G Yagüe-Guirao ^1,11^, PL Valero-Guillen ^1,12^, FV Aviles-Plaza ^1,13^, J Cabezas-Herrera ^1,13^, A Martinez-Lopez ^1,13^, M Martinez-Villanueva ^1,13^, JA Noguera-Velasco ^1,13^, A Franco-Garcia ^1,14^, AM Garcia-Serna ^1,14^, T Hernandez-Caselles ^1,14,18^, E Martin-Orozco ^1,14,18^, M Norte-Munoz ^1,14^, M Canovas ^1,14^, E Cantero-Cano ^1^, T de Diego ^1,14^, JM Pastor ^1,14^, RA Sola-Martínez ^1,14^, A Esteban-Gil ^1,17^, JT Fernandez-Breis ^1,15^, MV Alcantara ^16^, S Hernandez ^16^, C Lopez-Soler ^16^. ^1^ Biomedical Research Institute of Murcia (IMIB-Arrixaca), Murcia, Spain; ^2^ Paediatric Respiratory Unit, “Virgen de la Arrixaca” Children’s University Clinical Hospital, University of Murcia, Spain; ^3^ Department of Public Health Sciences, University of Murcia, Spain; ^4^ Department of Paediatrics, University of Murcia, Spain; ^5^ Obstetrics & Gynaecology Service, “Virgen de la Arrixaca” University Clinical Hospital, University of Murcia, Spain; ^6^ Food Science and Technology Department, Faculty of Veterinary, University of Murcia, Spain; ^7^ Department of Physiology, University of Murcia, Spain; ^8^ Regional Atmospheric Modelling Group, Department of Physics, University of Murcia, Spain; ^9^ Department of Public Health Sciences, University of Murcia, Spain; ^10^ Microbiology Service, General University Hospital Consortium, University of Valencia, Spain; ^11^ Microbiology Service, University Clinical Hospital “Virgen de la Arrixaca”, University of Murcia, Spain; ^12^ Microbiology and Genetics Department, University of Murcia, Spain; ^13^ Molecular Therapy and Biomarkers Research Group, Clinical Analysis Service, University Clinical Hospital “Virgen de la Arrixaca”, University of Murcia, Spain; ^14^ Department of Biochemistry and Molecular Biology B and Immunology, University of Murcia, Spain; ^15^ Department of Informatics and Systems, University of Murcia, Spain; ^16^ Paediatric and Adolescent Clinical Psychology University Research Group (GUIIA-PC), University of Murcia, Spain; ^17^ Foundation for Healthcare Training & Research of the Region of Murcia (FFIS); ^18^ Network of Asthma and Adverse and Allergic Reactions (ARADyAL).

## References

[1] Martinez M. (1992). Tissue levels of polyunsaturated fatty acids during early human development. J. Pediatr..

[2] Dobbing J., Sands J. (1973). Quantitative growth and development of human brain. Arch. Dis. Child..

[3] Innis S.M. (2000). The role of dietary n-6 and n-3 fatty acids in the developing brain. Dev. Neurosci..

[4] Lauritzen L., Brambilla P., Mazzocchi A., Harslof L.B., Ciappolino V., Agostoni C. (2016). DHA Effects in Brain Development and Function. Nutrients.

[5] Gil-Sanchez A., Koletzko B., Larque E. (2012). Current understanding of placental fatty acid transport. Curr. Opin. Clin. Nutr. Metab. Care.

[6] Koletzko B., Cetin I., Brenna J.T. (2007). Dietary fat intakes for pregnant and lactating women. Br. J. Nutr..

[7] EFSA Panel on Dietetic Products, Nutrition, and Allergies (NDA) (2010). Scientific opinion of the Panel on Dietary Reference Values for fats, including saturated fatty acids, polyunsaturated fatty acids, monounsaturated fatty acids, *trans* fatty acids, and cholesterol. EFSA J..

[8] Cinelli G., Fabrizi M., Rava L., Ciofi Degli Atti M., Vernocchi P., Vallone C., Pietrantoni E., Lanciotti R., Signore F., Manco M. (2016). Influence of Maternal Obesity and Gestational Weight Gain on Maternal and Foetal Lipid Profile. Nutrients.

[9] Thomas B.A., Ghebremeskel K., Lowy C., Offley-Shore B., Crawford M.A. (2005). Plasma fatty acids of neonates born to mothers with and without gestational diabetes. Prostaglandins Leukot. Essent. Fatty Acids.

[10] Wijendran V., Bendel R.B., Couch S.C., Philipson E.H., Cheruku S., Lammi-Keefe C.J. (2000). Fetal erythrocyte phospholipid polyunsaturated fatty acids are altered in pregnancy complicated with gestational diabetes mellitus. Lipids.

[11] Min Y., Lowy C., Ghebremeskel K., Thomas B., Bitsanis D., Crawford M.A. (2005). Fetal erythrocyte membrane lipids modification: Preliminary observation of an early sign of compromised insulin sensitivity in offspring of gestational diabetic women. Diabet. Med..

[12] Pagan A., Prieto-Sanchez M.T., Blanco-Carnero J.E., Gil-Sanchez A., Parrilla J.J., Demmelmair H., Koletzko B., Larque E. (2013). Materno-fetal transfer of docosahexaenoic acid is impaired by gestational diabetes mellitus. Am. J. Physiol. Endocrinol. Metab..

[13] Gazquez A., Prieto-Sanchez M.T., Blanco-Carnero J.E., Ruiz-Palacios M., Nieto A., van Harskamp D., Oosterink J.E., Schierbeek H., van Goudoever J.B., Demmelmair H. (2019). Altered materno-fetal transfer of 13C-polyunsaturated fatty acids in obese pregnant women. Clin. Nutr..

[14] Dube E., Gravel A., Martin C., Desparois G., Moussa I., Ethier-Chiasson M., Forest J.C., Giguere Y., Masse A., Lafond J. (2012). Modulation of fatty acid transport and metabolism by maternal obesity in the human full-term placenta. Biol. Reprod..

[15] Prieto-Sanchez M.T., Ruiz-Palacios M., Blanco-Carnero J.E., Pagan A., Hellmuth C., Uhl O., Peissner W., Ruiz-Alcaraz A.J., Parrilla J.J., Koletzko B. (2017). Placental MFSD2a transporter is related to decreased DHA in cord blood of women with treated gestational diabetes. Clin. Nutr..

[16] Segura M.T., Demmelmair H., Krauss-Etschmann S., Nathan P., Dehmel S., Padilla M.C., Rueda R., Koletzko B., Campoy C. (2017). Maternal BMI and gestational diabetes alter placental lipid transporters and fatty acid composition. Placenta.

[17] Ortega-Senovilla H., Alvino G., Taricco E., Cetin I., Herrera E. (2009). Gestational diabetes mellitus upsets the proportion of fatty acids in umbilical arterial but not venous plasma. Diabetes Care.

[18] Djelmis J., Ivanisevic M., Desoye G., van Poppel M., Berberovic E., Soldo D., Oreskovic S. (2018). Higher Cord Blood Levels of Fatty Acids in Pregnant Women With Type 1 Diabetes Mellitus. J. Clin. Endocrinol. Metab..

[19] Min Y., Djahanbakhch O., Hutchinson J., Eram S., Bhullar A.S., Namugere I., Ghebremeskel K. (2016). Efficacy of docosahexaenoic acid-enriched formula to enhance maternal and fetal blood docosahexaenoic acid levels: Randomized double-blinded placebo-controlled trial of pregnant women with gestational diabetes mellitus. Clin. Nutr..

[20] Monthe-Dreze C., Penfield-Cyr A., Smid M.C., Sen S. (2018). Maternal Pre-Pregnancy Obesity Attenuates Response to Omega-3 Fatty Acids Supplementation During Pregnancy. Nutrients.

[21] Morales M., Alcantara-Lopez M.V., Cabezas-Herrera J.A., De Diego T., Hernnadez-Caselles T., Jimenez-Guerrero P., Larque E., Lopez-Soler C., Martinez-Gracia C., Martinez-Torres A. (2021). The Nutrition in Early Life and Asthma (NELA) birth cohort study: Rationale, design, and methods. Paediatr. Perinat. Epidemiol..

[22] National Diabetes Data Group (1979). Classification and diagnosis of diabetes mellitus and other categories of glucose intolerance. Diabetes.

[23] Carrascosa A., Fernandez J.M., Fernandez C., Ferrandez A., Lopez-Siguero J.P., Sanchez E., Sobradillo B., Yeste D. (2008). Spanish growth studies 2008. New anthropometric standards. Endocrinol. Nutr..

[24] WHO (2008). Training Course on Child Growth Assessment. WHO Child Growth Standards.

[25] Vioque J., Navarrete-Muñoz E.-M., Gimenez-Monzó D., García-de-la-Hera M., Granado F., Young I.S., Ramón R., Ballester F., Murcia M., Rebagliato M. (2013). Reproducibility and validity of a food frequency questionnaire among pregnant women in a Mediterranean area. Nutr. J..

[26] Haytowitz D.B., Ahuja J.K.C., Wu X., Somanchi M., Nickle M., Nguyen Q.A., Roseland J.N.M., Williams J.R., Patterson K.Y., Li Y. USDA National Nutrient Database for Standard Reference, Legacy Release. https://data.nal.usda.gov/dataset/usda-national-nutrient-database-standard-reference-legacy-release.

[27] Leth T., Ovesen L., Hansen K. (1998). Fatty acid composition of meat from ruminants, with special emphasis on trans fatty acids. J. Am. Oil Chem. Soc..

[28] Larque E., Garaulet M., Pérez-Llamas F., Zamora S., Tebar F. (2003). Fatty acid composition and nutritional relevance of most widely consumed margarines in Spain. Grasas Aceites.

[29] Vicario I.M., Griguol V., León-Camacho M. (2003). Multivariate Characterization of the Fatty Acid Profile of Spanish Cookies and Bakery Products. J. Agric. Food Chem..

[30] Fernandez-San Juan P.M. (2009). Transfatty acids (tFA): Sources and intake levels, biological effects and content in commercial Spanish food. Nutr. Hosp..

[31] Willett W. (2013). Nutritional Epidemiology.

[32] Folch J., Lees M., Sloane Stanley G.H. (1957). A simple method for the isolation and purification of total lipides from animal tissues. J Biol. Chem..

[33] Stoffel W., Chu F., Ahrens E. (1959). Analysis of long-chain fatty acids by gas liquid chromatography. Micromethod for preparation of methyl esters. Anal. Chem..

[34] Domingo-Salvany A., Regidor E., Alonso J., Alvarez-Dardet C. (2000). Proposal for a social class measure. Working Group of the Spanish Society of Epidemiology and the Spanish Society of Family and Community Medicine. Aten Primaria.

[35] Tomedi L.E., Chang C.C., Newby P.K., Evans R.W., Luther J.F., Wisner K.L., Bodnar L.M. (2013). Pre-pregnancy obesity and maternal nutritional biomarker status during pregnancy: A factor analysis. Public Health Nutr..

[36] Vidakovic A.J., Gishti O., Voortman T., Felix J.F., Williams M.A., Hofman A., Demmelmair H., Koletzko B., Tiemeier H., Jaddoe V.W. (2016). Maternal plasma PUFA concentrations during pregnancy and childhood adiposity: The Generation R Study. Am. J. Clin. Nutr..

[37] Al-Otaibi H., Hussein N., Mustafa H., Al-Mudaires N. (2020). Obesity, gestational weight gain, and polyunsaturated fatty acids profile in pregnant Saudi women. Bioinformation.

[38] Calder P.C. (2010). Omega-3 fatty acids and inflammatory processes. Nutrients.

[39] Aye I.L., Lager S., Ramirez V.I., Gaccioli F., Dudley D.J., Jansson T., Powell T.L. (2014). Increasing maternal body mass index is associated with systemic inflammation in the mother and the activation of distinct placental inflammatory pathways. Biol. Reprod..

[40] Sibbons C.M., Brenna J.T., Lawrence P., Hoile S.P., Clarke-Harris R., Lillycrop K.A., Burdge G.C. (2014). Effect of sex hormones on n-3 polyunsaturated fatty acid biosynthesis in HepG2 cells and in human primary hepatocytes. Prostaglandins Leukot. Essent. Fatty Acids.

[41] Kitson A.P., Marks K.A., Shaw B., Mutch D.M., Stark K.D. (2013). Treatment of ovariectomized rats with 17beta-estradiol increases hepatic delta-6 desaturase enzyme expression and docosahexaenoic acid levels in hepatic and plasma phospholipids. Prostaglandins Leukot. Essent. Fatty Acids.

[42] Christian L.M., Young A.S., Mitchell A.M., Belury M.A., Gracious B.L., Arnold L.E., Fristad M.A. (2017). Body weight affects omega-3 polyunsaturated fatty acid (PUFA) accumulation in youth following supplementation in post-hoc analyses of a randomized controlled trial. PLoS ONE.

[43] Yee L.D., Lester J.L., Cole R.M., Richardson J.R., Hsu J.C., Li Y., Lehman A., Belury M.A., Clinton S.K. (2010). Omega-3 fatty acid supplements in women at high risk of breast cancer have dose-dependent effects on breast adipose tissue fatty acid composition. Am. J. Clin. Nutr..

[44] Helland I.B., Saugstad O.D., Smith L., Saarem K., Solvoll K., Ganes T., Drevon C.A. (2001). Similar effects on infants of n-3 and n-6 fatty acids supplementation to pregnant and lactating women. Pediatrics.

[45] Connor W.E., Lowensohn R., Hatcher L. (1996). Increased docosahexaenoic acid levels in human newborn infants by administration of sardines and fish oil during pregnancy. Lipids.

[46] Velzing-Aarts F.V., van der Klis F.R., van der Dijs F.P., van Beusekom C.M., Landman H., Capello J.J., Muskiet F.A. (2001). Effect of three low-dose fish oil supplements, administered during pregnancy, on neonatal long-chain polyunsaturated fatty acid status at birth. Prostaglandins Leukot. Essent. Fatty Acids.

[47] Ortega-Senovilla H., Schaefer-Graf U., Herrera E. (2020). Pregnant women with gestational diabetes and with well controlled glucose levels have decreased concentrations of individual fatty acids in maternal and cord serum. Diabetologia.

[48] Ogundipe E., Samuelson S., Crawford M.A. (2020). Gestational diabetes mellitus prediction? A unique fatty acid profile study. Nutr. Diabetes.

[49] Zhu Y., Li M., Rahman M.L., Hinkle S.N., Wu J., Weir N.L., Lin Y., Yang H., Tsai M.Y., Ferrara A. (2019). Plasma phospholipid n-3 and n-6 polyunsaturated fatty acids in relation to cardiometabolic markers and gestational diabetes: A longitudinal study within the prospective NICHD Fetal Growth Studies. PLoS Med..

[50] Leveille P., Rouxel C., Plourde M. (2018). Diabetic pregnancy, maternal and fetal docosahexaenoic acid: A review of existing evidence. J. Matern. Fetal Neonatal. Med..

[51] Herrera E., Ortega-Senovilla H. (2010). Disturbances in lipid metabolism in diabetic pregnancy—Are these the cause of the problem?. Best Pract. Res. Clin. Endocrinol. Metab..

[52] Ruiz-Palacios M., Ruiz-Alcaraz A.J., Sanchez-Campillo M., Larque E. (2017). Role of Insulin in Placental Transport of Nutrients in Gestational Diabetes Mellitus. Ann. Nutr. Metab..

