# Supplementary material for: Self-Reported DHA Supplementation during Pregnancy and Its Association with Obesity or Gestational Diabetes in Relation to DHA Concentration in Cord and Maternal Plasma: Results from NELA, a Prospective Mother-Offspring Cohort
Source: Nutrients. 2021 Mar 4;13(3):843. doi: 10.3390/nu13030843 (PMC8000695; doi:10.3390/nu13030843)
Supplement: Supplementary file 1 [file nutrients-13-00843-s001.pdf]

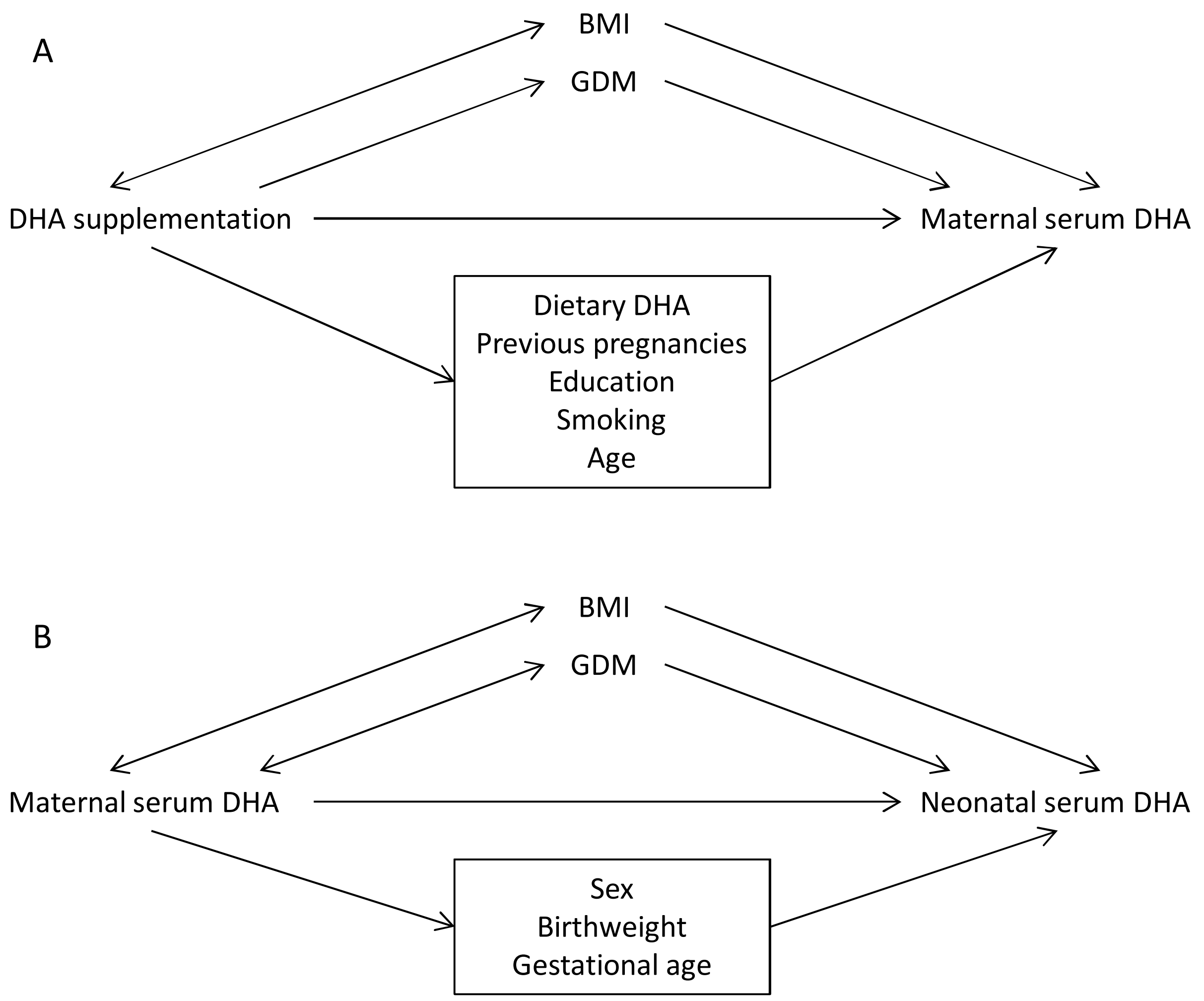

Figure S1. Directed acyclic graph (DAG) used to define potential covariates (box) affecting maternal (A) and neonatal serum DHA (B). BMI, body mass index; GDM, gestational diabetes mellitus.
